# The role of serum uric acid in survival prediction in patients with acute myocardial infarction accompanied by heart failure with preserved ejection fraction

**DOI:** 10.3389/fcvm.2025.1622275

**Published:** 2025-11-10

**Authors:** Soohyun Kim, Kwan Yong Lee, Kyung An Kim, Jaeho Byeon, Sanghoon Shin, Byung-Hee Hwang, Jin Jin Kim, Eun Ho Choo, Chan Joon Kim, Young Kyoung Sa, Mahn-Won Park, Sangho Hyun, Andrew H. Yoon, Youngkeun Ahn, Kiyuk Chang

**Affiliations:** 1 Cardiovascular Center and Cardiology Division, Incheon St. Mary’s Hospital, Catholic University of Korea, Seoul, Republic of Korea; 2 Cardiovascular Center and Cardiology Division, Seoul St. Mary’s Hospital, Catholic University of Korea, Seoul, Republic of Korea; 3 Cardiovascular Research Institute for Intractable Disease, College of Medicine, Catholic University of Korea, Seoul, Republic of Korea; 4 Cardiovascular Center and Cardiology Division, Uijeongbu St. Mary’s Hospital, Catholic University of Korea, Uijeonbu, Republic of Korea; 5 Cardiovascular Center and Cardiology Division, Yeouido St. Mary’s Hospital, Catholic University of Korea, Seoul, Republic of Korea; 6 Cardiovascular Center and Cardiology Division, Daejeon St. Mary’s Hospital, Catholic University of Korea, Daejeon, Republic of Korea; 7 College of Medicine, Catholic University of Korea, Seoul, Republic of Korea; 8 Department of Cardiology, Cardiovascular Center, Chonnam National University Hospital, Gwangju, Republic of Korea

**Keywords:** heart failure with preserved ejection fraction (HFpEF), uric acid, hyperuricemia, acute myocardial infarction, heart failure

## Abstract

**Background:**

Heart failure with preserved ejection fraction (HFpEF) is defined as presenting with clinical symptoms and signs of heart failure with a concomitant left ventricular ejection fraction ≥50%. However, the prognostic role of serum uric acid in HFpEF is not well understood.

**Methods:**

In total, 757 patients with HFpEF and acute myocardial infarction were included in the analysis. Hyperuricemia was defined as a serum uric acid level >6.9 mg/dL in men and >5.4 mg/dL in women at the time of diagnosis of acute myocardial infarction. The primary outcome was all-cause mortality.

**Results:**

Among the enrolled patients, 164 and 593 were placed into the high uric acid and normal uric acid groups, respectively. After a median follow up of 4.8 years [interquartile range: 3.2–7.1], 54 (32.9%) in the high serum uric acid group and 92 (15.5%) in the normal serum uric acid group had died. Hyperuricemia was independently associated with all-cause mortality (*p* < 0.001) and cardiovascular death [73 (12.3%) vs. 44 (26.8%); *p* < 0.001]. The increased risk of mortality remained consistent in the multivariate Cox proportional hazards model (hazard ratio: 1.5; 95% confidence interval: 1.03–2.19; *p* = 0.033). After classifying the enrolled patients according to their Heart Failure Association-Pre-test assessment, Echocardiography and natriuretic peptide, Functional testing, and Final etiological work-up (HFA-PEFF) score (366 with a HFA-PEFF score <3 and 391 with a HFA-PEFF score ≥3), hyperuricemia was also found to be associated with all-cause mortality in patients with a score greater than the intermediate score (≥3 points) (*p* < 0.001).

**Conclusions:**

In a cohort with acute myocardial infarction, hyperuricemia was independently associated with all-cause mortality in patients with HFpEF.

**Clinical Trial Registration:**

ClinicalTrials.gov, COREA-AMI NCT02806102.

## Introduction

Heart failure (HF) is a complex clinical syndrome that consists of cardinal symptoms and signs that are a result of structural or functional abnormalities of the heart, and can be divided into distinct phenotypes based on the measurement of the left ventricular ejection fraction (LVEF) (1). Heart failure with preserved ejection fraction (HFpEF) is defined by symptoms and signs of heart failure with an LVEF of 50% or greater and a structural or functional abnormality of the heart with the presence of left ventricular diastolic dysfunction or raised filling pressure (1). HFpEF is heterogenous and caused by multiple mechanisms, including cardiac aging and cardiometabolic disorders, and the incidence of HFpEF has increased due to population aging and coexisting conditions (2). Despite the increasing incidence and prevalence of HFpEF, no therapeutic approach has been established to modify disease progression or reduce mortality in HFpEF. In this context, plasma natriuretic peptide (BNP) and N-terminal plasma natriuretic peptide (NT-proBNP) remain the only biomarkers with both diagnostic and prognostic value. Hence, a novel method of screening and evaluating underlying cardiovascular diseases and cardiovascular risk factors is necessary because an appropriate strategy for HFpEF has not yet been established (3).

Serum uric acid (SUA) has been validated for its association with cardiovascular disease. Various studies have demonstrated a correlation between hyperuricemia and other comorbidities, including hypertension, diabetes mellitus, metabolic syndrome, chronic kidney disease, coronary artery disease, and heart failure (4–6). However, the prognostic role of SUA in HF has not been well investigated, and its relationship with HFpEF has scarcely been investigated.

Ischemic heart disease and myocardial infarction (MI) are the most common etiologies of HF, especially heart failure with reduced ejection fraction (HFrEF), but they also contribute to the development of HFpEF (7). In addition, to aid in making the challenging diagnosis of HFpEF, a few score-based algorithms [Heavy (BMI >30), Hypertensive (≥2 antihypertensive meds), atrial Fibrillation, Pulmonary hypertension (PASP >35 mmHg), Elder (age >60), Filling pressure (E/e′ > 9) (H_2_FPEF) and Heart Failure Association-Pre-test assessment, Echocardiography and natriuretic peptide, Functional testing, and Final etiological work-up (HFA-PEFF)] have emerged as diagnostic tools (8, 9). We investigated the prognostic role of SUA in patients with HFpEF after an acute MI. In addition, we used SUA and the HFA-PEFF score to predict the mortality and cardiovascular outcomes in patients with HFpEF related to an acute MI.

## Methods

### Study protocols and population selection

The Convergent REgistry of cAtholic and chonnAm University for Acute Myocardial Infarction (COREA-AMI) was designed to evaluate real-world, long-term clinical outcomes in all consecutive patients with an acute MI at nine major cardiac centers in Korea. The COREA-AMI is a single registry that prospectively enrolled patients with an acute MI undergoing percutaneous coronary intervention (PCI). It was organized in two phases: COREA-AMI I (January 2004–December 2009) and COREA-AMI II (January 2010–August 2014). The latter phase not only extended the follow-up of the former cohort but also included additional patient enrollment. In this study, both phases were analyzed as one integrated registry. The clinical, angiographic, and follow-up data of all patients with an acute MI were consecutively registered in the electronic, web-based case report system. The COREA-AMI study was conducted in accordance with the Declaration of Helsinki. This observational study was approved by the institutional review board of our institution (IRB No. XC15RSMI0089K) and performed in accordance with the Strengthening the Reporting of Observational Studies in Epidemiology (STROBE) guidelines (10). The COREA-AMI registry is registered on ClinicalTrials.gov (study ID: NCT02806102).

**Figure 1:**
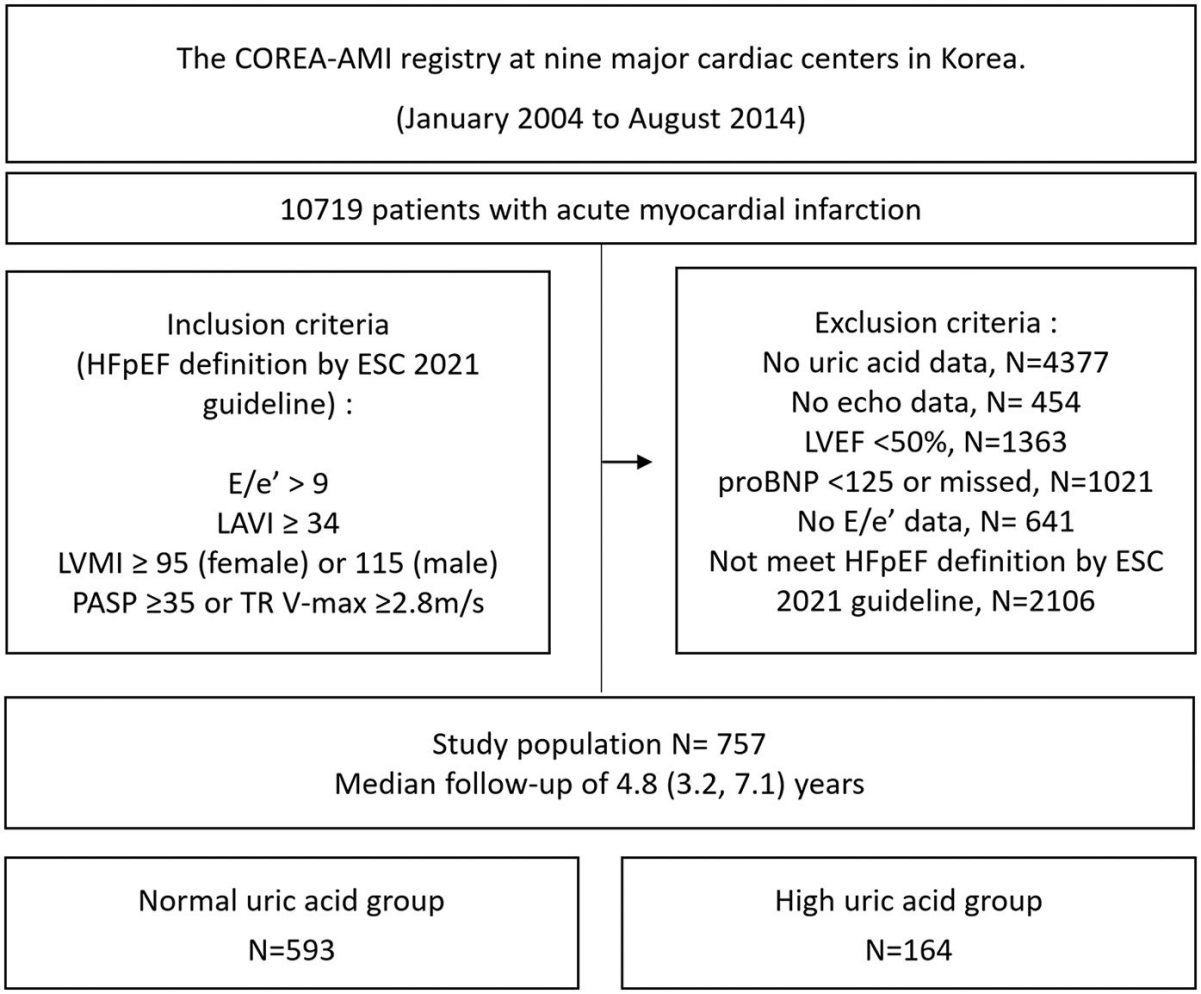
Study flow chart. HFpEF, heart failure with preserved ejection fraction; LAVI, left atrial volume index; LVMI, left ventricular mass index; PASP, pulmonary artery systolic pressure; TR, tricuspid regurgitation; LVEF, left ventricular ejection fraction; NT-proBNP, N-terminal pro B-type natriuretic peptide.

Of the 10,719 patients with an acute MI who underwent PCI with drug-eluting stents (DES) in this registry, we excluded any patients who did not undergo serum uric acid testing (*N* = 4,377) or echocardiography (*N* = 454) at baseline. Patients with an LVEF <50% in their baseline echocardiography (*N* = 1,363) were also excluded. Among the remaining patients, we excluded those who did not fulfill the following HFpEF criteria for left ventricular diastolic dysfunction or increased left ventricular filling pressure according to the 2021 European Society of Cardiology (ESC) HF guideline: E/eʹ at rest over 9, left atrial volume index (LAVI) over 34, left ventricular mass index (LVMI) ≥95 g/m^2^ for women and ≥115 g/m^2^ for men, NT-proBNP >125 pg/mL, pulmonary arterial systolic pressure ≥35, and tricuspid regurgitation (TR) velocity ≥2.8 m/s (*N* = 3,786). The size of the final enrolled study population was 757. The study flow chart is depicted in Figure 1. The patients were divided into a high SUA group (uric acid >6.9 mg/dL for men and >5.4 mg/dL for women) or a normal SUA group using their initial serum uric acid level measured at the time of the acute MI. Transthoracic echocardiography was performed at each center and descriptions of the measured values were left by the physicians at each center. Guideline-directed medical therapy was conducted by each physician. The HFA-PEFF diagnostic algorithm is a stepwise scoring system that incorporates functional, morphological, and biomarker domains, with a total score ranging from 0 to 6. When the score is higher than 5 points, an immediate diagnosis of HFpEF is recommended. With a score of 2–4 points, a diastolic stress test or invasive hemodynamic measurements are recommended for a definitive diagnosis of HFpEF, whereas a score of ≤1 makes HFpEF very unlikely (8). In our study, the patients were categorized using an HFA-PEFF score cutoff of ≥3 as an intermediate-to-high probability of HFpEF. The HFA-PEFF score was calculated retrospectively for risk stratification purposes and not as a primary inclusion criterion.

### Study outcomes

The primary endpoint of the study was all-cause mortality. The secondary outcome was all-cause mortality according to the HFA-PEFF score.

### Statistical analysis

The baseline demographics, medications at discharge, laboratory results, and angiographic characteristics are presented as mean ± standard deviation (SD) or median and interquartile range (IQR), based on the normal distribution of these continuous variables. Differences in the continuous variables between the groups were evaluated using Student's *t*-test for independent samples or the Mann–Whitney *U* test. The categorical data are presented as frequencies and proportions and were compared using the chi-squared (*χ*^2^) test (or Fisher's exact test when any expected count was <5 for a 2 × 2 table). The optimal cut-off SUA value in our study was determined using receiver operating characteristic (ROC) curve analysis with the Youden index. The thresholds for hyperuricemia in our study were >6.9 mg/dL for men and >5.4 mg/dL for women **(**Supplementary Figure 1**)**.

The cumulative event rates in each group were calculated using a Kaplan–Meier estimator and compared using the log-rank statistic. Because differences in the baseline characteristics could significantly affect outcomes, sensitivity analyses were performed to adjust for confounders as much as possible. As a sensitivity analysis, propensity score (PS)-matched cohorts were constructed for patients with normal vs. high uric acid using 1:1 matching and the nearest-neighbor method without replacement on the propensity scores obtained from logistic regression with a caliper width of 0.1. In accordance with established guidelines for propensity score analysis, matching was conducted using all available baseline covariates to ensure adequate control for potential confounding (11). To identify predictors of events, we used a multivariable Cox proportional hazards model. We predefined the covariates by including both clinically relevant factors known to be important based on prior studies and the baseline characteristics that were significantly different between the two groups (those with a *p*-value of <0.05 in the univariable analysis). The adjusted variables for the multivariate Cox proportional hazards regression analysis were age ≥65, sex, diabetes mellitus (DM), hypertension, history of stroke, Killip class, estimated glomerular filtration rate (eGFR) <30, chronic lung disease, angiotensin-converting enzyme (ACE) inhibitors or angiotensin Ⅱ receptor blocker (ARB), oral anticoagulants, hemoglobin, creatinine, triglyceride, high-density lipoprotein (HDL), and multivessel disease. The balance between the two groups after PS-matching or inverse probability treatment weighted (IPTW) adjustment was assessed by calculating the percent standardized mean differences with a threshold of 0.2. The percent standardized mean differences after propensity-score matching were within ±10% across all the matched covariates, demonstrating successful achievement of balance between the comparative groups. Each measure was conducted using R version 4.5 (R Foundation for Statistical Computing, Vienna, Austria). Statistical significance was indicated by a two-tailed *p-*value < 0.05.

## Results

### Baseline characteristics

The baseline clinical characteristics of the 757 patients are listed in Table 1. The mean age among the enrolled patients was 63.5 ± 12.2 years, whereas 355 (46.9%) were older than 65 years and 220 (29.1%) were female. Of the enrolled patients, 164 (21.7%) were classified into the high SUA group, while 593 (78.3%) were classified into the normal SUA group. The mean SUA level obtained at baseline was 8.0 ± 2.9 mg/dL in the high SUA group and 4.7 ± 1.1 mg/dL in the normal SUA group. The prevalence of DM, dyslipidemia, atrial fibrillation on baseline electrocardiogram, and previous stroke was similar in both groups. High SUA was associated with a higher Global Registry of Acute Coronary Events (GRACE) score, higher NT-proBNP, lower HDL cholesterol, lower hemoglobin, higher serum creatinine, and renal dysfunction. Among the enrolled patients, 453 (59.8%) had been diagnosed with an ST-segment elevation myocardial infarction (STEMI). More than 50% of the patients had multivessel coronary artery disease that included more than two vessels. Regarding the characteristics of the PCI procedures, neither the total stent number nor the length or generation of the stent was different between the two groups (Table 2). The mean HFA-PEFF score was significantly higher in the high SUA group (2.8 ± 1.2 in the high SUA group vs. 2.5 ± 1.2 in the normal SUA group; *p* = 0.001) and the proportion of patients with a HFA-PEFF score ≥3 was higher in the high SUA group [100 (61.0%) vs. 291 (49.1%); *p* = 0.009]. Echocardiographic measures are presented in Table 3. The mean LVEF was 59.6 ± 6.0% in the high SUA group and 58.7 ± 6.0% in the normal SUA group (*p* = 0.088). There were no statistical differences in the other echocardiographic values between the groups. The PS distribution and the baseline characteristics after PS-matching and the IPTW analysis are presented in Supplementary Tables 1–2.

**Table 1:** Baseline clinical and laboratory characteristics.

Variable	Total patients	High uric acid group	Normal uric acid group	*p*-Value
	(*N* = 757)	(*N* = 164)	(*n* = 593)	
Age, years	63.5 ± 12.2	64.9 ± 13.6	63.1 ± 11.8	0.139
Age ≥65	355 (46.9)	87 (53.0)	268 (45.2)	0.09
Female	220 (29.1)	61 (37.2)	159 (26.8)	0.013
BMI	24.1 ± 3.0	24.5 ± 3.0	24.0 ± 3.0	0.077
DM	278 (36.7)	66 (40.2)	212 (35.8)	0.335
Hypertension	423 (55.9)	107 (65.2)	316 (53.3)	0.008
Dyslipidemia	163 (21.5)	37 (22.6)	126 (21.2)	0.799
History of stroke	51 (6.7)	15 (9.1)	36 (6.1)	0.224
Current smoker	305 (40.3)	55 (33.5)	250 (42.2)	0.057
Previous MI	17 (2.2)	1 (0.6)	16 (2.7)	0.141
Previous PCI	43 (5.7)	7 (4.3)	36 (6.1)	0.489
Previous CABG	4 (0.5)	1 (0.6)	3 (0.5)	1
Atrial fibrillation on baseline ECG	19 (2.5)	6 (3.7)	13 (2.2)	0.27
eGFR < 30 mL/min/1.73 m^2^	34 (4.5)	13 (2.2)	21 (12.8)	<0.001
Chronic liver disease	12 (1.6)	1 (0.6)	11 (1.9)	0.479
Chronic lung disease	13 (1.7)	4 (2.4)	9 (1.5)	0.494
Cancer	30 (4.0)	11 (6.7)	19 (3.2)	0.07
Killip class ≥2	142 (18.8)	48 (29.3)	94 (15.9)	<0.001
LVEF	58.8 ± 6.0	59.6 ± 6.0	58.7 ± 6.0	0.088
Laboratory findings				
Uric acid	5.4 ± 2.1	8.0 ± 2.9	4.7 ± 1.1	<0.001
CK-MB, peak, ng/mL	128.9 ± 131.5	130.4 ± 132.6	123.7 ± 127.9	0.557
proBNP, mg/dL	1,768.8 ± 4,973.1	2,862.7 ± 5,896.7	1,466.3 ± 4,646.1	0.006
Hemoglobin, mg/dL	13.9 ± 2.1	13.4 ± 2.5	14.0 ± 1.9	0.006
Creatinine, mg/dL	1.1 ± 0.8	1.5 ± 1.2	1.0 ± 0.6	<0.001
eGFR, mL/min/1.73 m^2^	189 (25.0)	87 (53.0)	102 (17.2)	<0.001
High-sensitivity CRP, mg/dL	3.0 ± 13.8	3.2 ± 8.6	2.9 ± 15.0	0.771
Total cholesterol, mg/dl	175.3 ± 41.0	175.1 ± 41.6	175.4 ± 40.9	0.94
Triglyceride, mg/dL	114.2 ± 92.1	128.6 ± 120.8	110.2 ± 82.1	0.067
High-density lipoprotein, mg/dL	40.1 ± 10.9	37.3 ± 11.6	40.9 ± 10.5	<0.001
Low-density lipoprotein, mg/dL	110.4 ± 35.7	109.7 ± 36.6	110.5 ± 35.4	0.799

Data are presented as the *n* (%) for categorical variables. Continuous variables are presented as the mean ± standard deviation or median (Q1, Q3), according to whether they were normally distributed or not. The potent P2Y12 inhibitors include ticagrelor or prasugrel. BMI, body mass index; DM, diabetes mellitus; HTN, hypertension; MI, myocardial infarction; PCI, primary coronary intervention; CABG, coronary artery bypass graft; ECG, electrocardiography; LVEF, left ventricular ejection fraction; eGFR, estimated glomerular filtration rate; BNP, brain natriuretic peptide; CK-MB, creatinine kinase MB isoenzyme; HbA1c, hemoglobin A1C; CRP, C-reactive protein; ACEi; angiotensin-converting enzyme inhibitors; ARB, angiotensin II receptor blockers; DAPT, dual antiplatelet therapy.

**Table 2:** Procedural characteristics and medications prescribed after percutaneous coronary intervention.

Procedural characteristics and medications	Total patients	High uric acid group	Normal uric acid group	*p*-Value
	(*N* = 757)	(*N* = 164)	(*n* = 593)	
ST-segment elevation MI	453 (59.8)	98 (59.8)	355 (59.9)	1
HFA-PEFF score	2.5 ± 1.2	2.8 ± 1.3	2.5 ± 1.2	0.001
HFA-PEFF score ≥3	391 (51.7)	100 (61.0)	291 (49.1)	0.009
Medication at discharge				
Aspirin	748 (98.8)	161 (98.2)	587 (99.0)	0.416
Clopidogrel	618 (81.6)	142 (86.6)	476 (80.3)	0.083
Ticagrelor	74 (9.8)	11 (6.7)	63 (10.6)	0.178
Prasugrel	63 (8.3)	11 (6.7)	52 (8.8)	0.493
Potent P2Y12 inhibitor	137 (18.1)	22 (13.4)	115 (19.4)	0.1
Beta-blocker	673 (88.9)	147 (89.6)	526 (88.7)	0.845
ACEi or ARB	566 (74.8)	118 (72.0)	448 (75.5)	0.403
Oral anticoagulant	17 (2.2)	7 (4.3)	10 (1.7)	0.069
Statin at discharge	722 (95.4)	154 (93.9)	568 (95.8)	0.42
Angiographic characteristics				
Multivessel disease	430 (56.8)	103 (62.8)	327 (55.1)	0.096
Left main PCI	21 (2.8)	3 (1.8)	18 (3.0)	0.592
Left anterior descending PCI	411 (54.3)	78 (47.6)	333 (56.2)	0.062
Left circumflex PCI	193 (25.5)	36 (22.0)	157 (26.5)	0.282
Right coronary artery PCI	356 (47.0)	88 (53.7)	268 (45.2)	0.067
Total stent number	1.6 ± 0.9	1.5 ± 0.9	1.6 ± 0.9	0.4
Total stent length	37.8 ± 24.2	37.7 ± 24.5	37.8 ± 24.2	0.969
Bifurcation PCI with two stents	9 (1.2)	2 (1.2)	7 (1.2)	1
Long stenting >60 mm	29 (3.8)	5 (3.0)	24 (4.0)	0.719
Restenosis lesion	8 (1.1)	1 (0.6)	7 (1.2)	1
Ostial lesion	22 (2.9)	2 (1.2)	20 (3.4)	0.192
Second generation DES	552 (72.9)	119 (72.6)	433 (73.0)	0.986

Data are presented as the *n* (%) for categorical variables. Continuous variables are presented as the mean ± standard deviation or median (Q1, Q3), according to whether they were normally distributed or not. The potent P2Y12 inhibitors include ticagrelor or prasugrel. MI, myocardial infarction; HFA-PEFF score, Heart Failure Association-Pre-test assessment, Echocardiography and natriuretic peptide, Functional testing, and Final etiology score; PCI, primary coronary intervention; ACEi, angiotensin-converting enzyme inhibitors; ARB, angiotensin II receptor blockers; DES, drug-eluting stent.

**Table 3:** Baseline echocardiographic parameters.

Parameter	Total patients (*N* = 757)	High uric acid group (*N* = 164)	Normal uric acid group (*N* = 593)	*p*-Value
Left ventricular ejection fraction (%)	58.8 ± 6.0	59.6 ± 6.0	58.7 ± 6.0	0.088
Left ventricular end-systolic diameter (mm)	38.8 ± 8.5	31.0 ± 6.9	30.8 ± 8.9	0.784
Left ventricular end-diastolic diameter (mm)	47.9 ± 7.7	48.1 ± 6.2	47.9 ± 8.1	0.78
Left ventricular end-systolic volume (mL)	33.4 ± 11.4	32.4 ± 10.9	33.7 ± 11.5	0.289
Left ventricular end-diastolic volume (mL)	78 ± 21.2	78.5 ± 20.9	77.9 ± 21.2	0.786
LA diameter	36.4 ± 7.2	38.2 ± 6.6	35.9 ± 7.3	0.001
Left atrial volume index (mL/m^2^)	29.7 ± 16.8	31.5 ± 15.1	29.2 ± 17.2	0.422
Estimated PASP (mmHg)	31.6 ± 10.3	33.4 ± 12.8	31.1 ± 9.5	0.152
E/e'	13.0 ± 8.2	13.8 ± 5.5	12.7 ± 8.8	0.05

PASP, pulmonary artery systolic pressure.

### Clinical outcomes

**Figure 2:**
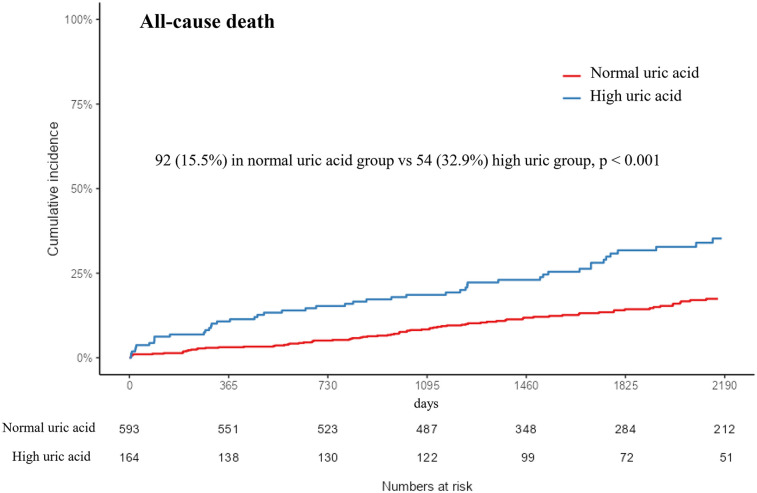
Kaplan–Meier event curve of the cumulative incidence of all-cause mortality according to uric acid level. SUA, serum uric acid.

During the median follow-up time of 4.8 years [IQR 3.2; 7.1], 92 and 54 patients died in the normal SUA and high SUA groups, respectively. The Kaplan–Meier analysis showed that hyperuricemia was associated with increased risk for all-cause mortality [92 (15.5%) vs. 54 (32.9%); *p* < 0.001] (Figure 2). The difference in the result was mainly driven by a higher number of cardiac deaths in the high SUA group [73 (12.3%) vs. 44 (26.8%); *p* < 0.001]. The multivariate Cox proportional hazards model showed increased risk of all-cause mortality in the high SUA group [hazard ratio (HR): 1.5; 95% confidence interval (CI): 1.03–2.19; *p* = 0.033] (Supplementary Table 3). High SUA was also associated with increased risk of readmission for heart failure in the multivariate analysis (HR: 3.74; 95% CI: 1.33–10.5; *p* = 0.012). There were no significant differences in the event rates of readmission for unstable angina, recurrent MI, stent thrombosis, revascularization, and ischemic stroke (*p* = 0.394, 0.239, 0.322, 0.631, and 0.633, respectively). Concordant results were shown in the sensitivity analyses, including multivariate adjustment, PS matching, and IPTW adjustment (Table 4). The multivariate Cox regression demonstrated that age ≥65, diabetes mellitus, cancer, and NT-proBNP were significant predictors of all-cause mortality after adjustment (*p* < 0.05, respectively) (Supplementary Table 3).

**Table 4:** Cumulative outcomes according to uric acid level in patients with HFpEF and AMI.

Outcome					Unadjusted		Multivariable-adjusted		PS-matched cohort		IPTW	
	Total	High uric acid group	Normal uric acid group	*p*-Value	HR (95% CI)	*p*-Value†	HR (95% CI)	*p*-Value	HR (95% CI)	*p*-Value	HR (95% CI)	*p*-Value
	(*n* = 757)	(*n* = 164)	(*n* = 593)									
All-cause mortality	146 (19.3)	54 (32.9)	92 (15.5)	<0.001	2.36 (1.68–3.3)	<0.001	1.50 (1.03–2.19)	0.033	2.03 (1.25–3.30)	0.004	2.39 (1.27–4.49)	0.007
Cardiac death	117 (15.5)	44 (26.8)	73 (12.3)	<0.001	2.43 (1.67–3.53)	<0.001	1.52 (1.00–2.32)	0.05	2.02 (1.19–3.43)	0.009	2.42 (1.15–5.10)	0.02
Readmission for heart failure	19 (2.5)	9 (5.5)	10 (1.7)	0.01	3.71 (1.50–9.14)	0.004	3.74 (1.33–10.50)	0.012	2.84 (0.73–11.09)	0.134	3.74 (1.1–12.69)	0.035
Readmission for unstable angina	73 (9.6)	14 (8.5)	59 (9.9)	0.694	0.94 (0.52–1.68)	0.835	0.76 (0.41–1.42)	0.394	0.96 (0.46–2.01)	0.91	0.69 (0.33–1.42)	0.313
Recurrent MI	32 (4.2)	5 (3.0)	27 (4.6)	0.53	0.74 (0.28–1.91)	0.529	0.53 (0.19–1.52)	0.239	0.80 (0.26–2.54)	0.711	0.87 (0.23–3.36)	0.842
Definite or probable ST-elevation MI	12 (1.6)	1 (0.6)	11 (1.9)	0.479	0.36 (0.05–2.79)	0.328	0.34 (0.04–2.85)	0.322	0.21 (0.02–1.77)	0.151	0.20 (0.02–1.63)	0.133
Revascularization	107 (14.1)	20 (12.2)	87 (14.7)	0.497	0.89 (0.55–1.44)	0.631	0.78 (0.47–1.31)	0.354	1.19 (0.64–2.24)	0.579	0.76 (0.41–1.38)	0.365
Ischemic stroke	27 (3.6)	8 (4.9)	19 (3.2)	0.432	1.65 (0.72–3.77)	0.235	1.25 (0.50–3.08)	0.633	1.31 (0.40–4. 29)	0.656	0.75 (0.32–1.80)	0.524

PS, propensity score; IPTW, inverse probability treatment weighted; SMD, standardized mean differences; HR, hazard ratio; MI, myocardial infarction.

### Risk prediction and stratification

**Figure 3:**
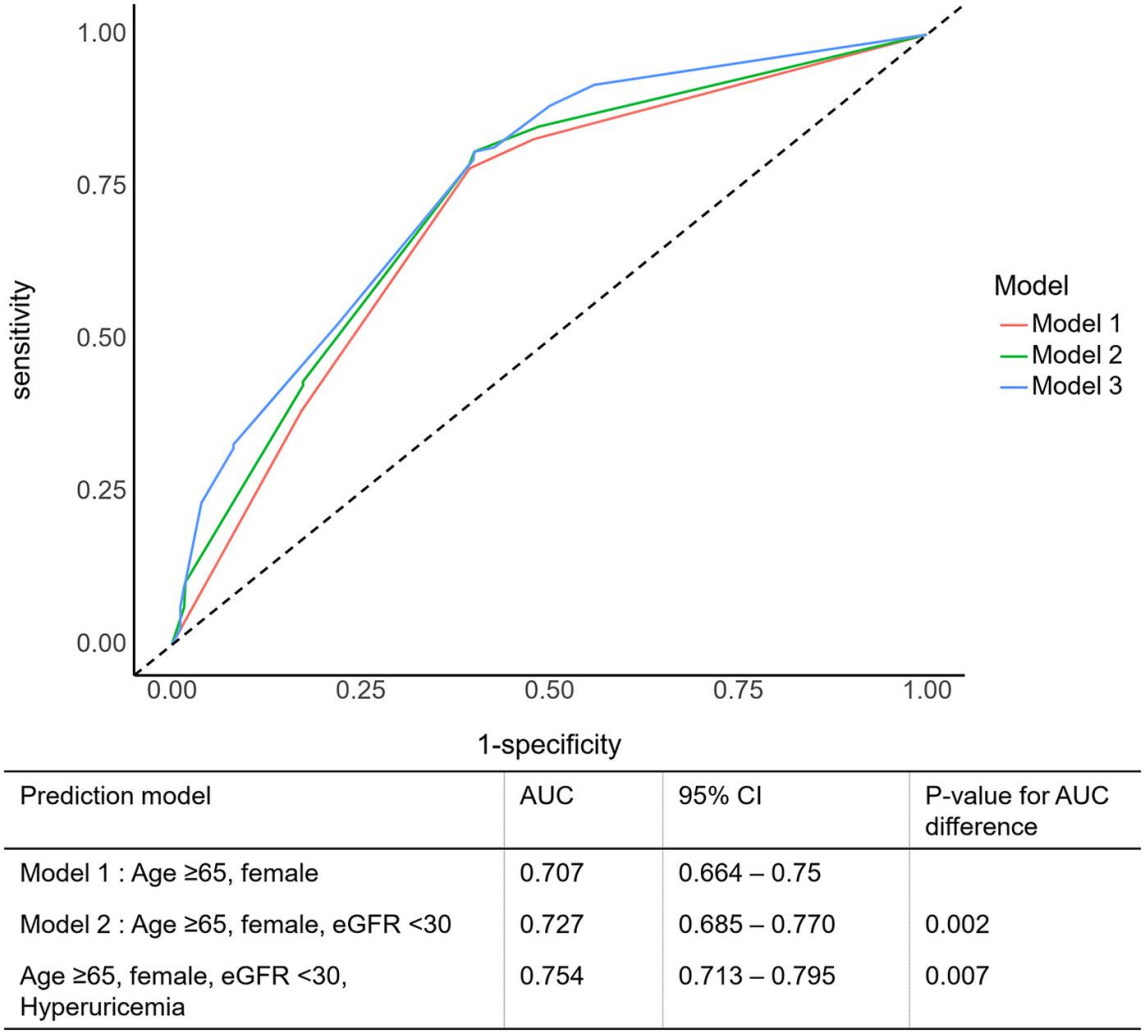
Prognostic impact of serum uric acid level in patients with an acute myocardial infarction. AUC, area under the ROC curve; CI, confidence interval; eGFR, estimated glomerular filtration rate.

A ROC curve analysis was performed to evaluate the ability of the serum uric acid level to predict mortality in patients with acute MI and HFpEF. The optimal cutoff value in our study was determined using a ROC curve analysis with the Youden index, and the association with long-term mortality was evaluated in a large multicenter acute MI cohort. The sensitivity and specificity of the serum uric acid levels at a cutoff of >6.9 mg/dL (410.41 µmol/L) were 34.94% and 83.7% for men, respectively, and, at a cut-off of >5.4 mg/dL (321.19 µmol/L), 39.68% and 77.07% for women, respectively. The addition of a high SUA to age >65, sex, and eGFR <30, which are well-validated conventional cardiovascular risk factors in acute MI, significantly increased the discriminant ability of the model to predict mortality compared with the conventional risk factors alone with an area under the curve (AUC) of 0.754 (95% CI: 0.713–0.797, *p* = 0.007) (Figure 3). The addition of high SUA to the conventional risk factors enabled significantly higher discrimination and reclassification abilities for all-cause mortality than the conventional risk factors alone.

### Subgroup analysis

**Figure 4:**
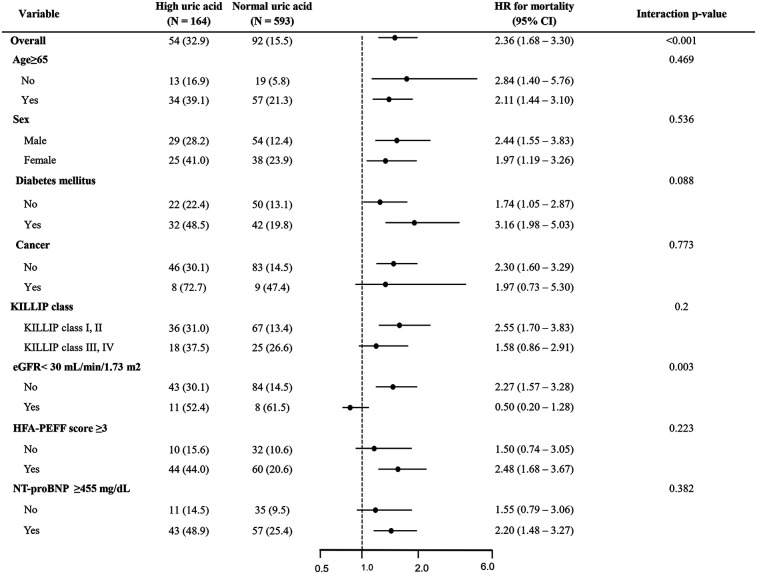
Forest plot for all-cause death. HR, hazard ratio; CI, confidence interval; eGFR, estimated glomerular filtration rate; NT-pro BNP, N-terminal pro B-type natriuretic peptide.

In the subgroup analysis, high uric acid levels were significantly associated with increased mortality across the majority of the groups (Figure 4). This association was particularly evident in the patients aged 65 and older (HR = 2.11; 95% CI 1.44–3.10), men (HR = 2.44; 95% CI 1.55–3.83), those with diabetes (HR = 3.16; 95% CI 1.98–5.03), patients with relatively preserved renal function (eGFR ≥ 30; HR = 2.27; 95% CI 1.57–3.28), and those with HFA-PEFF scores of 3 or higher (HR = 2.48; 95% CI 1.68–3.67). Conversely, no significant association between uric acid levels and mortality was observed in the high-risk groups, such as those with eGFR below 30 or those in Killip class III/IV.

## Discussion

In this large, nationwide registry-based study, we investigated the prognostic role of SUA in patients with HFpEF after an acute MI. The main findings of our study were that (i) elevated SUA levels were independently associated with increased all-cause mortality, primarily driven by cardiac death; (ii) the optimal SUA cut-off for mortality in patients with HFpEF and an acute MI was >6.9 mg/dL in men and >5.4 mg/dL in women; (iii) the predictive power of SUA was enhanced when added to the conventional risk factors, supporting its incremental prognostic role; (iv) in our subgroup analysis, a high SUA level was strongly associated with increased mortality, especially in patients with higher HFA-PEFF scores (≥3), while the association was attenuated in the patients with advanced renal dysfunction (eGFR <30). These results suggest that SUA is a biomarker that provides valuable prognostic information in patients with HFpEF and an acute MI, although its utility may not be applicable in patients with advanced chronic kidney disease (CKD).

The potential prognostic role of SUA is supported by several mechanisms. Serum uric acid is the end-product of purine metabolism and is metabolized by xanthine oxidase. An elevated serum uric acid level reflects increased xanthine oxidase activity, which is associated with enhanced oxidative stress, endothelial dysfunction, and microvascular inflammation, leading to systemic hypertension with vascular dysfunction and ultimately contributing to HF progression (12–15). These processes are central to the pathophysiology of HFpEF, which is characterized by cardiac remodeling with impaired ventricular relaxation and diastolic dysfunction (16, 17).

Although SUA has been recognized as a biomarker that is associated with cardiovascular morbidity and mortality, most prior studies have focused on general heart failure populations or those with reduced ejection fraction. Hyperuricemia has been consistently linked to oxidative stress, endothelial dysfunction, and adverse hemodynamic effects in HFrEF, and an elevated SUA level has been correlated with cardiovascular outcomes. Previous studies showed a correlation between uric acid level and HF. However, HFpEF is a heterogeneous syndrome with a complex pathophysiology distinct from HFrEF, and therefore, findings from studies on HFrEF cannot be generalized to HFpEF populations. A meta-analysis of nearly 42,000 patients from 28 studies on the association between SUA and HF outcomes found that every 1 mg/dL increase in SUA level was associated with a 19% higher odds of developing HF and a 4% higher risk of all-cause mortality (18). However, these studies did not distinguish between HF phenotypes. Prior research from our group of more than 5,000 patients also demonstrated that a high SUA level is an independent risk factor for all-cause mortality in patients with an acute MI, with better prognostic value than conventional risk factors, without differentiating HFpEF (19).

HFpEF is highly prevalent, accounting for up to 50% of HF etiology, and is associated with significant morbidity and mortality, and while the total incidence of HF has decreased, the incidence of HFpEF has increased (20). To date, only a few studies have examined the prognostic role of SUA in HFpEF, and these studies have been limited by short follow-up durations or a lack of clinical outcomes. Little has been revealed about the association between HFpEF and SUA. Carnicelli et al. found that hyperuricemia was associated with adverse biomarker profiles, including NT-proBNP and impaired cardiac function measured by exercise capacity during 24 weeks of a relatively short-term follow-up (21). In a retrospective study of 1,009 patients with hypertension and echocardiographic signs of diastolic dysfunction, an elevated SUA level was independently associated with an incident of new-onset HFpEF over a median of 7.2 years, suggesting a role for SUA in the development of this disease, with each 1 mg/dL increase predicting a 10.7% higher risk (22). Our study, with more than 700 well-characterized patients followed for approximately 5 years, is one of the first large-scale analyses to establish a significant long-term association between SUA and mortality specifically in patients with HFpEF after an MI.

One of the important findings of our study is the distinction between SUA as a prognostic marker in general HFpEF populations vs. in patients with advanced CKD. The PARAGON-HF trial showed that hyperuricemia was associated with increased risk of HF hospitalization and cardiovascular death; however, patients with eGFR <30 were excluded (23). In our cohort, although fewer than 5% of the patients had eGFR <30, this subgroup showed no significant association between SUA and mortality. Co-existence of CKD and HFpEF has been widely investigated, with CKD affecting half of HFpEF patients due to their similar underlying morbidities and sharing co-risk factors and microvascular dysfunction (24). Since SUA levels often increase due to impaired renal clearance, this complicates the interpretation of this finding in patients with HFpEF, in whom renal dysfunction is common. In patients with advanced CKD, competing risk factors and impaired clearance may outweigh the effect of SUA on mortality. In contrast, in patients with preserved renal function, SUA appears to reflect systemic metabolic and inflammatory pathways that directly contribute to adverse outcomes in HFpEF. This distinction emphasizes the need to interpret SUA levels in the context of renal function and indicates that patients with eGFR <30 should be regarded as a distinct subgroup with unique pathophysiology and competing risks. The interplay between SUA, renal function, and cardiovascular risk remains complex. Our findings may help clarify this relationship by showing that elevated SUA levels predicted higher mortality in patients with eGFR ≥30, while the association was absent in patients with advanced renal dysfunction.

Our study has several important clinical implications. SUA is an inexpensive, widely available laboratory measure that can be easily incorporated into routine practice. Due to HFpEF being challenging to diagnose, several diagnostic scoring systems, including the HFA-PEFF score, have been suggested for risk stratification. It has been shown that in patients with an acute MI, a higher HFA-PEFF score was associated with adverse clinical outcomes (25). In our study, SUA levels were significantly associated with increased mortality across the majority of the groups, with this trend being particularly pronounced in the patients with higher HFA-PEFF scores (≥3). As a higher HFA-PEFF score indicates a greater likelihood of HFpEF, our findings provided additional prognostic information beyond the conventional risk factors. The mean HFA-PEFF score in our study was 2.5 ± 1.2, indicating that some of the patients may have had a score of 1, which could imply the exclusion of HFpEF. However, HFpEF was diagnosed based on guideline-directed echocardiographic and biomarker criteria at baseline. This discrepancy reflects the heterogeneity of diagnostic algorithms in real-world practice. Furthermore, our study findings suggest that SUA could serve as a useful adjunctive biomarker for identifying high-risk patients with HFpEF after an MI, especially in settings where advanced biomarkers or imaging may not be readily available. Nevertheless, whether lowering SUA improves outcomes in patients with HFpEF remains uncertain. Information regarding uric acid-lowering therapies, such as allopurinol, was not available in our registry, and prior trials on HFrEF have been largely neutral, with no evidence of better outcomes in patients with HFpEF. Sacubitril/valsartan, which has uricosuric properties, was associated with reduced HF hospitalization in PARAGON-HF, but it is unclear whether this effect was mediated by uric acid lowering. Sub-analyses suggested improved quality of life and renal function after lowering SUA levels, but causality remains unproven (26, 27). Another novel HF medication, sodium-glucose cotransporter-2 (SGLT2) inhibitors, lowers serum uric acid levels by suppressing its production and enhancing excretion. However, the association between the uric acid-lowering effect and the cardioprotection of SGLT2 inhibitors does not appear to be causal, but rather reflects an independent process that shares a common pathophysiological mechanism (28, 29). In this context, our findings indicate that an elevated SUA level can serve as a prognostic biomarker, but its reduction alone may not necessarily have clinical implications. Future studies are warranted to incorporate SUA into clinical decision-making and clarify whether interventions targeting SUA directly can modify outcomes in patients with HFpEF.

## Study limitations

Several limitations of our study should be acknowledged. First, this was an observational registry-based study, where confounding cannot be excluded despite adjustment and propensity-score matching. Second, the patients’ SUA levels were only measured at baseline, and we lacked data on temporal changes or treatment of hyperuricemia. Third, information on gout and uric acid-lowering medications was not available, preventing any evaluation of the therapeutic implications. Fourth, our study cohort was derived from an acute MI population rather than an HFpEF registry, where symptoms of HF were not collected. This also raises the possibility that our findings may not be fully generalizable to non-ischemic patients with HFpEF. Nevertheless, the patients fulfilled the echocardiographic criteria for HFpEF as recommended by current guidelines. Fifth, we excluded approximately 40% of eligible patients for whom baseline SUA measurements were missing, which could have led to selection bias. Finally, the proportion of women in our cohort was relatively low compared with other studies on HFpEF, which conflicts with the fact that HFpEF is more prevalent in women. This may be attributable to the fact that our study was derived from an AMI cohort, which was primarily composed of patients with ischemic heart disease.

## Conclusion

In conclusion, our study demonstrated that an elevated serum uric acid level is independently associated with increased long-term all-cause mortality in patients with HFpEF following an acute MI. The association was most pronounced in the patients with higher HFA-PEFF scores and preserved renal function, while no significant relationship was observed in those with advanced CKD, highlighting the importance of patient characteristics when interpreting serum uric acid levels. Our study also underscores the potential role of serum uric acid as an easily accessible prognostic biomarker in HFpEF and provides a basis for future research on whether targeting uric acid may improve outcomes in select patient populations.

## Data Availability

The datasets are not readily available because they contain patient-level clinical information that is restricted under institutional and ethical regulations. Requests to access these datasets should be directed to Kwan Yong Lee, cycle0210@gmail.com.
