# Supplementary material for: The role of serum uric acid in survival prediction in patients with acute myocardial infarction accompanied by heart failure with preserved ejection fraction
Source: Front Cardiovasc Med. 2025 Nov 10;12:1622275. doi: 10.3389/fcvm.2025.1622275 (PMC12641431; doi:10.3389/fcvm.2025.1622275)

Supplementary Figure 1. Receiver-operating characteristic (ROC) curves of serum uric acid for predicting all-cause mortality in patients with HFpEF following acute myocardial infarction. (A) Male patients. (B) Female patients. Optimal cut-off values were derived using the Youden index.

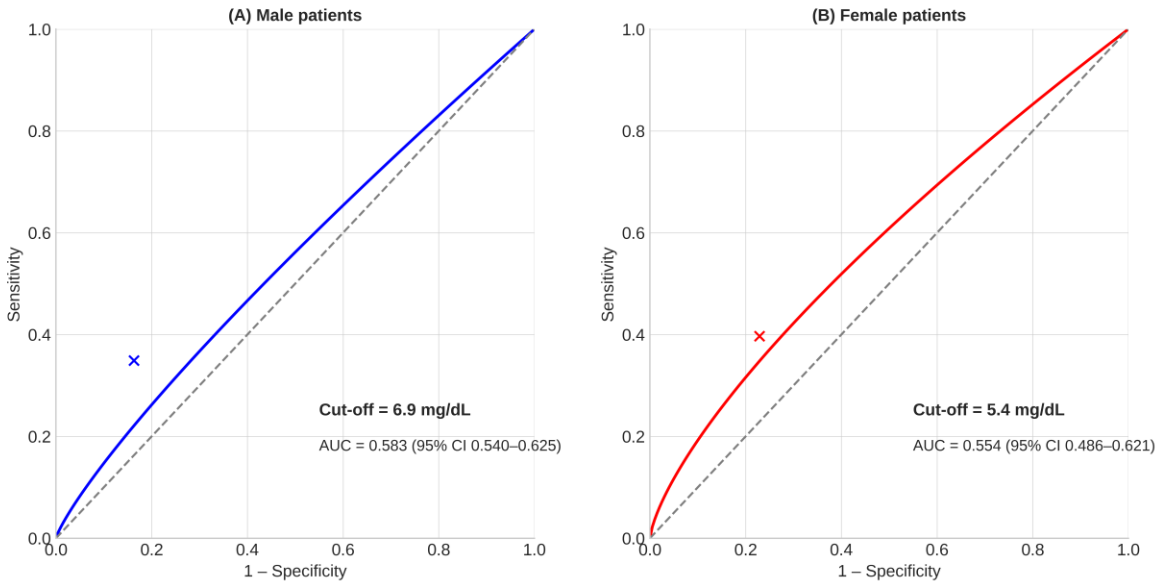

Supplement: Supplementary file 1 [file Image1.pdf]
