# Supplementary material for: The role of serum uric acid in survival prediction in patients with acute myocardial infarction accompanied by heart failure with preserved ejection fraction
Source: Front Cardiovasc Med. 2025 Nov 10;12:1622275. doi: 10.3389/fcvm.2025.1622275 (PMC12641431; doi:10.3389/fcvm.2025.1622275)
Supplement: Supplementary file 2 [file Datasheet1.pdf]

Supplementary table 1. Baseline clinical and medical characteristics after propensity-score matching.

	Original cohort					PS matched cohort					IPTW cohort				
	Total (n=757)	High uric acid (n=164)	Normal uric acid (n=593)	p-value	SMD	Total (n=296)	High uric acid (n=148)	Normal uric acid (n=148)	p-value	SMD	Total (n=1549.4)	High uric acid (n=761.5)	Normal uric acid (n=787.9)	p-value	SMD
**Clinical characteristics**															
Age, yr	63.5 ± 12.2	64.9 ± 13.6	63.1 ± 11.8	0.139	0.136	64.1 ± 12.7	64.6 ± 13.5	63.6 ± 11.9	0.509	0.077	63.4 ± 12.3	63.2 ± 12.7	63.5 ± 11.8	0.832	0.022
Age ≥65	355 (46.9)	87 (53.0)	268 (45.2)	0.09	0.158	148 (50.0)	76 (51.4)	72 (48.6)	0.727	0.054	668.3 (43.1)	309.1 (39.2)	359.3 (47.2)	0.234	0.161
Female	220 (29.1)	61 (37.2)	159 (26.8)	0.013	0.224	106 (35.8)	52 (35.1)	54 (36.5)	0.904	0.028	526.4 (34.0)	302.0 (38.3)	224.5 (29.5)	0.265	0.188
BMI	24.1 ± 3.0	24.5 ± 3.0	24.0 ± 3.0	0.077	0.157	24.1 ± 3.0	24.3 ± 2.9	23.9 ± 3.1	0.215	0.144	24.2 ± 3.0	24.3 ± 2.9	24.0 ± 3.0	0.31	0.111
DM	278 (36.7)	66 (40.2)	212 (35.8)	0.335	0.093	106 (35.8)	55 (37.2)	51 (34.5)	0.716	0.056	595.2 (38.4)	321.9 (40.9)	273.3 (35.9)	0.537	0.102
Hypertension	423 (55.9)	107 (65.2)	316 (53.3)	0.008	0.245	194 (65.5)	94 (63.5)	100 (67.6)	0.541	0.085	932.1 (60.2)	499.5 (63.4)	432.6 (56.8)	0.326	0.135
Dyslipidemia	163 (21.5)	37 (22.6)	126 (21.2)	0.799	0.032	63 (21.3)	32 (21.6)	31 (20.9)	1	0.017	334.9 (21.6)	176.4 (22.4)	158.4 (20.8)	0.753	0.039
History of Stroke	51 (6.7)	15 (9.1)	36 (6.1)	0.224	0.116	29 (9.8)	14 (9.5)	15 (10.1)	1	0.023	99.2 (6.4)	46.0 (5.8)	53.2 (7.0)	0.611	0.046
Current smoker	305 (40.3)	55 (33.5)	250 (42.2)	0.057	0.178	107 (36.1)	52 (35.1)	55 (37.2)	0.809	0.042	580.5 (37.5)	277.3 (35.2)	303.2 (39.8)	0.482	0.095
Previous MI	17 (2.2)	1 (0.6)	16 (2.7)	0.141	0.164	3 (1.0)	0 (0.0)	3 (2.0)	0.247	0.203	21.4 (1.4)	2.1 (0.3)	19.2 (2.5)	0.008	0.193
Previous PCI	43 (5.7)	7 (4.3)	36 (6.1)	0.489	0.081	15 (5.1)	5 (3.4)	10 (6.8)	0.289	0.154	84.5 (5.5)	33.8 (4.3)	50.7 (6.7)	0.477	0.104
Previous CABG	4 (0.5)	1 (0.6)	3 (0.5)	1	0.014	1 (0.3)	0 (0.0)	1 (0.7)	1	0.117	6.3 (0.4)	2.1 (0.3)	4.2 (0.6)	0.529	0.045
Atrial fibrillation on baseline ECG	19 (2.5)	6 (3.7)	13 (2.2)	0.27	0.087	10 (3.4)	6 (4.1)	4 (2.7)	0.748	0.075	42.5 (2.7)	22.1 (2.8)	20.4 (2.7)	0.942	0.008

eGFR <30 mL/min/1.73 m2	34 (4.5)	13 (2.2)	21 (12.8)	<0.001	0.411		9 (6.1)	12 (8.1)	0.651	0.079	71.2 (4.6)	36.7 (4.8)	34.5 (4.4)	0.815	0.021
Chronic liver disease	12 (1.6)	1 (0.6)	11 (1.9)	0.479	0.113	4 (1.4)	1 (0.7)	3 (2.0)	0.622	0.117	18.0 (1.2)	4.1 (0.5)	13.9 (1.8)	0.197	0.122
Chronic lung disease	13 (1.7)	4 (2.4)	9 (1.5)	0.494	0.066	6 (2.0)	3 (2.0)	3 (2.0)	1	<0.001	21.2 (1.4)	8.5 (1.1)	12.8 (1.7)	0.481	0.052
Cancer	30 (4.0)	11 (6.7)	19 (3.2)	0.07	0.162	17 (5.7)	9 (6.1)	8 (5.4)	1	0.029	66.2 (4.3)	30.9 (3.9)	35.3 (4.6)	0.73	0.035
KILLIP ≥2	142 (18.8)	48 (29.3)	94 (15.9)	<0.001	0.325	75 (25.3)	40 (27.0)	35 (23.6)	0.593	0.078	264.0 (17.0)	125.4 (15.9)	138.6 (18.2)	0.553	0.061
LVEF	58.8 ± 6.0	59.6 ± 6.0	58.7 ± 6.0	0.088	0.151	59.4 ± 5.9	59.2 ± 5.7	59.7 ± 6.1	0.409	0.096	58.6 ± 6.0	58.4 ± 6.1	58.9 ± 6.0	0.65	0.081
ST-segment elevation MI	453 (59.8)	98 (59.8)	355 (59.9)	1	0.002	178 (60.1)	88 (59.5)	90 (60.8)	0.906	0.028	976.8 (63.0)	523.2 (66.4)	453.6 (59.6)	0.274	0.142
HFA-PEFF score	2.5 ± 1.2	2.8 ± 1.3	2.5 ± 1.2	0.001	0.293	2.7 ± 1.2	2.8 ± 1.2	2.6 ± 1.2	0.214	0.145	2.5 ± 1.3	2.4 ± 1.3	2.6 ± 1.3	0.362	0.143
HFA-PEFF score ≥3	391 (51.7)	100 (61.0)	291 (49.1)	0.009	0.241	171 (57.8)	88 (59.5)	83 (56.1)	0.638	0.068	746.8 (48.2)	348.5 (44.2)	398.3 (52.3)	0.253	0.162
**Medication at discharge**															
Aspirin	748 (98.8)	161 (98.2)	587 (99.0)	0.416	0.069	292 (98.6)	146 (98.6)	146 (98.6)	1	<0.001	1534.4 (99.0)	780.8 (99.1)	753.6 (99.0)	0.84	0.015
Clopidogrel	618 (81.6)	142 (86.6)	476 (80.3)	0.083	0.17	247 (83.4)	128 (86.5)	119 (80.4)	0.211	0.164	1250.3 (80.7)	631.5 (80.2)	618.8 (81.3)	0.83	0.028
Ticagrelor	74 (9.8)	11 (6.7)	63 (10.6)	0.178	0.14	22 (7.4)	10 (6.8)	12 (8.1)	0.825	0.052	175.0 (11.3)	98.4 (12.5)	76.6 (10.1)	0.549	0.077
Prasugrel	63 (8.3)	11 (6.7)	52 (8.8)	0.493	0.077	26 (8.8)	10 (6.8)	16 (10.8)	0.305	0.144	121.8 (7.9)	59.0 (7.5)	62.7 (8.2)	0.813	0.028
Potent P2Y12 inhibitor	137 (18.1)	22 (13.4)	115 (19.4)	0.1	0.162	48 (16.2)	20 (13.5)	28 (18.9)	0.27	0.147	296.7 (19.2)	157.4 (20.0)	139.3 (18.3)	0.741	0.043
Beta-blocker	673 (88.9)	147 (89.6)	526 (88.7)	0.845	0.03	262 (88.5)	131 (88.5)	131 (88.5)	1	<0.001	1387.8 (89.6)	710.4 (90.2)	677.4 (88.9)	0.739	0.04
ACEi or ARB	566 (74.8)	118 (72.0)	448 (75.5)	0.403	0.082	216 (73.0)	107 (72.3)	109 (73.6)	0.896	0.03	1097.9 (70.9)	522.5 (66.3)	575.4 (75.6)	0.249	0.204
Oral anticoagulant	17 (2.2)	7 (4.3)	10 (1.7)	0.069	0.152	10 (3.4)	5 (3.4)	5 (3.4)	1	<0.001	37.3 (2.4)	22.7 (2.9)	14.6 (1.9)	0.523	0.063
Statin at discharge	722 (95.4)	154 (93.9)	568 (95.8)	0.42	0.085	280 (94.6)	139 (93.9)	141 (95.3)	0.797	0.06	1469.9 (94.9)	741.0 (94.1)	728.8 (95.7)	0.534	0.075

Data are presented as the n (%) for categorical variables and as the mean ± standard deviation for continuous variables. eGFR = 141 ∗ min(Scr/κ,1)α ∗ max(Scr/κ, 1) − 1.209 ∗ 0.993 Age ∗ 1.018 (if female) ∗ 1.159 (if black). Scr is serum creatinine (mg/dL), κ is 0.7 for females and 0.9 for males, α is −0.329 for females and −0.411 for males, min indicates the minimum of Scr/κ or 1, and max indicates the maximum of Scr/κ or 1. PS indicates propensity score; IPTW, inverse probability treatment weighted; SMD, standardized mean differences; BMI, body mass index; DM, diabetes mellitus; MI, myocardial infarction; CABG, coronary artery bypass graft; ECG, electrocardiography; eGFR, estimated glomerular filtration rate; LVEF, left ventricle ejection fraction; ACEi, angiotensin-converting enzyme inhibitors; ARB, angiotensin II receptor blockers

Supplementary table 2. Baseline Laboratory and Angiographic Characteristics after Propensity-score matching.

	Original cohort					PS matched cohort					IPTW cohort				
	Total (n=757)	High uric acid (n=164)	Normal uric acid (n=593)	p-value	SMD	Total (n=296)	High uric acid (n=148)	Normal uric acid (n=148)	p-value	SMD	Total (n=1549.4)	High uric acid (n=761.5)	Normal uric acid (n=787.9)	p-value	SMD
**Laboratory findings**															
Uric acid	5.4 ± 2.1	8.0 ± 2.9	4.7 ± 1.1	<0.001	1.481	6.4 ± 2.5	7.9 ± 2.7	4.9 ± 1.1	<0.001	1.42	6.1 ± 2.2	7.5 ± 2.2	4.8 ± 1.1	<0.001	1.552
CK-MB, peak, ng/mL	128.9 ± 131.5	130.4 ± 132.6	123.7 ± 127.9	0.557	0.051	128.5 ± 129.5	129.0 ± 128.0	127.9 ± 131.4	0.939	0.009	133.7 ± 128.4	130.6 ± 133.2	136.7 ± 123.8	0.722	0.048
proBNP, mg/dL	1768.8 ± 4973.1	2862.7 ± 5896.7	1466.3 ± 4646.1	0.006	0.263	2660.3 ± 6401.2	2858.5 ± 6039.2	2462.2 ± 6758.6	0.595	0.062	1763.7 ± 4809.5	1674.7 ± 4102.4	1855.8 ± 5452.4	0.66	0.038
Hemoglobin, mg/dL	13.9 ± 2.1	13.4 ± 2.5	14.0 ± 1.9	0.006	0.263	13.7 ± 2.3	13.6 ± 2.3	13.8 ± 2.2	0.316	0.117	13.8 ± 2.1	13.7 ± 2.2	13.9 ± 2.0	0.449	0.091
Creatinine, mg/dL	1.1 ± 0.8	1.5 ± 1.2	1.0 ± 0.6	<0.001	0.493	1.3 ± 0.8	1.3 ± 1.0	1.2 ± 0.6	0.316	0.117	1.1 ± 0.9	1.1 ± 0.8	1.1 ± 1.0	0.458	0.1
eGFR, mL/min/1.73 m2	189 (25.0)	87 (53.0)	102 (17.2)	<0.001	0.81	133 (44.9)	73 (49.3)	60 (40.5)	0.161	0.177	400.2 (25.8)	211.7 (26.9)	188.6 (24.8)	0.665	0.048
high-sensitivity CRP, mg/dL	3.0 ± 13.8	3.2 ± 8.6	2.9 ± 15.0	0.771	0.022	3.6 ± 18.8	2.8 ± 8.4	4.4 ± 25.2	0.471	0.084	2.9 ± 13.7	2.5 ± 7.2	3.4 ± 18.0	0.447	0.064
Total cholesterol, mg/dL	175.3 ± 41.0	175.1 ± 41.6	175.4 ± 40.9	0.94	0.007	175.7 ± 40.4	177.2 ± 41.5	174.2 ± 39.4	0.525	0.074	178.4 ± 39.6	183.0 ± 37.8	173.5 ± 40.9	0.037	0.241
Triglyceride, mg/dL	114.2 ± 92.1	128.6 ± 120.8	110.2 ± 82.1	0.067	0.179	123.6 ± 91.7	119.0 ± 82.3	128.2 ± 100.2	0.391	0.1	115.2 ± 90.6	116.5 ± 93.3	113.8 ± 88.1	0.792	0.029
High-density lipoprotein, mg/dL	40.1 ± 10.9	37.3 ± 11.6	40.9 ± 10.5	<0.001	0.325	38.4 ± 11.2	37.7 ± 11.8	39.0 ± 10.6	0.354	0.108	40.3 ± 11.6	40.6 ± 12.5	40.0 ± 10.6	0.755	0.052
Low-density lipoprotein, mg/dL	110.4 ± 35.7	109.7 ± 36.6	110.5 ± 35.4	0.799	0.023	109.9 ± 34.5	112.1 ± 36.7	107.8 ± 32.0	0.291	0.123	110.4 ± 33.0	111.5 ± 30.9	109.1 ± 35.1	0.461	0.073
**Angiographic characteristics**															

Multivessel disease	430 (56.8)	103 (62.8)	327 (55.1)	0.096	0.156	178 (60.1)	93 (62.8)	85 (57.4)	0.406	0.111	945.9 (61.1)	519.9 (66.0)	426.1 (55.9)	0.124	0.207
Left main PCI	21 (2.8)	3 (1.8)	18 (3.0)	0.592	0.078	10 (3.4)	3 (2.0)	7 (4.7)	0.334	0.15	945.9 (61.1)	519.9 (66.0)	426.1 (55.9)	0.124	0.207
Left anterior descending PCI	411 (54.3)	78 (47.6)	333 (56.2)	0.062	0.173	139 (47.0)	72 (48.6)	67 (45.3)	0.641	0.068	771.2 (49.8)	362.5 (46.0)	408.7 (53.7)	0.295	0.153
Left circumflex PCI	193 (25.5)	36 (22.0)	157 (26.5)	0.282	0.106	71 (24.0)	32 (21.6)	39 (26.4)	0.414	0.111	441.0 (28.5)	245.2 (31.1)	195.9 (25.7)	0.521	0.12
Right coronary artery PCI	356 (47.0)	88 (53.7)	268 (45.2)	0.067	0.17	155 (52.4)	78 (52.7)	77 (52.0)	1	0.014	822.2 (53.1)	461.0 (58.5)	361.1 (47.4)	0.115	0.224
Total stent number	1.6 ± 0.9	1.5 ± 0.9	1.6 ± 0.9	0.4	0.073	1.6 ± 0.9	1.6 ± 0.9	1.6 ± 0.9	0.948	0.008	1.7 ± 1.0	1.8 ± 1.1	1.6 ± 0.9	0.304	0.263
Total stent length	37.8 ± 24.2	37.7 ± 24.5	37.8 ± 24.2	0.969	0.003	37.9 ± 24.2	38.8 ± 25.1	37.0 ± 23.2	0.525	0.074	41.4 ± 26.2	45.4 ± 27.6	37.3 ± 24.1	0.157	0.312
Bifurcation PCI with two stents	9 (1.2)	2 (1.2)	7 (1.2)	1	0.004	4 (1.4)	1 (0.7)	3 (2.0)	0.622	0.117	13.8 (0.9)	4.3 (0.5)	9.5 (1.2)	0.316	0.075
Long stenting >60mm	29 (3.8)	5 (3.0)	24 (4.0)	0.719	0.054	10 (3.4)	5 (3.4)	5 (3.4)	1	<0.001	49.0 (3.2)	18.4 (2.3)	30.6 (4.0)	0.287	0.096
Restenosis lesion	8 (1.1)	1 (0.6)	7 (1.2)	1	0.061	4 (1.4)	1 (0.7)	3 (2.0)	0.622	0.117	33.8 (2.2)	19.6 (2.5)	14.2 (1.9)	0.786	0.043
Ostial lesion	22 (2.9)	2 (1.2)	20 (3.4)	0.192	0.144	8 (2.7)	2 (1.4)	6 (4.1)	0.282	0.167	31.6 (2.0)	6.4 (0.8)	25.2 (3.3)	0.047	0.176
2nd generation DES	552 (72.9)	119 (72.6)	433 (73.0)	0.986	0.01	212 (71.6)	106 (71.6)	106 (71.6)	1	<0.001	1158.5 (74.8)	612.8 (77.8)	545.8 (71.7)	0.267	0.141

Supplemental Table 3. Predictive Factors for Mortality According to multivariable Cox Regression Analysis

	Unadjusted		Multivariable-adjusted	
	HR (95% CI)	p-value†	HR (95% CI)	p-value†
High uric acid	2.36 (1.68-3.30)	<0.001	1.5 (1.03-2.19)	0.033
Old age (>65)	4.73 (3.19-7.00)	<0.001	3.44 (2.24-5.30)	<0.001
Female	1.96 (1.41-2.72)	<0.001	0.94 (0.64-1.38)	0.748
Hypertension	1.88 (1.32-2.67)	<0.001	0.9 (0.61-1.33)	0.599
Diabetes mellitus	1.99 (1.44-2.75)	<0.001	1.85 (1.3-2.63)	<0.001
History of stroke	2.21 (1.35-3.63)	0.002	1.45 (0.88-2.4)	0.149
eGFR <30 mL/min/1.73 m^2	4.88 (3.00-7.92)	<0.001	0.84 (0.45-1.54)	0.571
Cancer	3.91 (2.35-6.50)	<0.001	2.29 (1.31-4.02)	0.004
KILLIP ≥2	1.81 (1.26-2.58)	0.001	1.01 (0.68-1.49)	0.98
Hemoglobin, mg/dL	0.77 (0.72-0.82)	<0.001	0.98 (0.89-1.08)	0.633
proBNP, mg/dL (Logarithmic form)	3.45 (2.75-4.32)	<0.001	2.81 (2.13-3.70)	<0.001
HDL cholesterol	0.98 (0.97-1.00)	0.02	1.00 (0.98-1.01)	0.666
Triglyceride	1.00 (1.00-1.00)	0.075	1.00 (1.00-1.00)	0.869
High HFA-PEFF score 3, 4 5	2.48 (1.73-3.55)	<0.001	0.69 (0.45-1.54)	0.571
